# Serological analysis of gluten-related antibodies in idiopathic neuropathies and cerebellar ataxia

**DOI:** 10.1007/s00415-025-13187-w

**Published:** 2025-06-07

**Authors:** Maxine D. Rouvroye, Janna Warendorf, Alexander Vrancken, Filip Eftimov, Luuk Wieske, Catharina G. Faber, Janneke G. J. Hoeijmakers, Jan Damoiseaux, Bart van de Warrenburg, Judith van Gaalen, Renate van der Molen, Gerd Bouma, Hetty Bontkes

**Affiliations:** 1 Department of Gastroenterology and Hepatology, AG&M Research Institute, Amsterdam UMC, Vrije Universiteit Amsterdam, Amsterdam, The Netherlands; 2 Department of Neurology, UMC Utrecht Brain Center, University Medical Center Utrecht, Utrecht, the Netherlands; 3 Department of Neurology, Amsterdam Neuroscience, Amsterdam UMC, University of Amsterdam, Amsterdam, The Netherlands; 4 Department of Neurology, Maastricht University Medical Center, Mental Health & Neuroscience Research Institute Maastricht, Maastricht, The Netherlands; 5 Central Diagnostic Laboratory, Maastricht University Medical Center, P. Debyelaan 25, 6229 HX Maastricht, The Netherlands; 6 Department of Neurology, Donders Institute for Brain, Cognition and Behaviour, Radboud University Medical Center, PO Box 9101, 6500 HB Nijmegen, The Netherlands; 7 Department of Neurology, Rijnstate Ziekenhuis, Velp, The Netherlands; 8 Department of Laboratory Medicine, Laboratory for Medical Immunology, Radboud University Medical Center, Nijmegen, the Netherlands; 9 Department of Laboratory Medicine, Laboratory Specialized Diagnostics and Research, Section Medical Immunology, Amsterdam Infection & Immunity, Amsterdam UMC, Vrije Universiteit Amsterdam, Amsterdam, The Netherlands

**Keywords:** Gluten, Ataxia, Small fibre neuropathy, Chronic idiopathic axonal polyneuropathy, Gliadin, Transglutaminase 6

## Abstract

Immune reactivity to gluten in the development of peripheral neuropathies and cerebellar ataxia has been suggested for decades, but evidence is scarce. The aim of the current study was to test the prevalence of tissue transglutaminase 2 (anti-TG2), tissue transglutaminase 6 (anti-TG6), and gliadin antibodies (anti-gliadin) in a large cross-sectional study. The sera of patients with idiopathic cerebellar ataxia, idiopathic small fibre neuropathy (SFN) and chronic idiopathic axonal polyneuropathy (CIAP), and controls with a comparable age distribution and men/women ratio were collected. The sera were analysed for anti-gliadin IgA/IgG (manufacturer’s and lower cut-off), anti-TG2 IgA and anti-TG6 IgA/IgG. In total, 683 samples were analysed: 476 patients (249 SFN, 161 CIAP and 66 idiopathic cerebellar ataxia) and 195 controls. There were no differences between disease and control group in the prevalence of elevated anti-TG6, anti-TG2 and anti-gliadin using the manufacturer’s cut-off. Using a lower cut-off of 3 U/mL, previously used by others for gluten-related neurological disorders, anti-gliadin IgA was positive in 20.8% patients vs 12.8% controls (*p* = 0.017) and anti-gliadin IgG in 7.6% vs 2.6% (*p* = 0.013), respectively. In subgroup analyses, significant differences were only observed in SFN for anti-gliadin IgA and in idiopathic cerebellar ataxia for anti-gliadin IgG using this lower cut-off after adjusting for sex and age. In conclusion, no difference in anti-TG2, anti-TG6 and anti-gliadin levels were observed between patients and controls. Only when using the lower cut-off (3 U/mL), the patients with SFN and idiopathic cerebellar ataxia were more often positive for anti-gliadin than controls. Whether these low-titre antibodies are gluten related, have any pathophysiological relevance, or reflect an epiphenomenon of neurodegeneration or gut inflammation is unknown.

## Introduction

In the last decades, the spectrum of conditions related to gluten ingestion has been extended to a wide range of extra-intestinal manifestations, including several neurological disorders [1]. These so called gluten-related neurological disorders (GRND) include peripheral neuropathy, cerebellar ataxia, myelopathy, and encephalopathy, among others. Gluten-related neurological disorders have been described to occur independently of intestinal symptoms and enteropathy [1]. In contrast to the well-established causative role of gluten for enteropathy in coeliac disease (CD), the relationship between ingested gluten and these neurological disorders is still debated. The possible underlying pathophysiological mechanisms are yet to be determined and serological studies have shown conflicting results [2–9]. Several potential serological markers have been suggested to support the diagnosis of GRND: anti-transglutaminase 2 (anti-TG2), anti-transglutaminase 6 (anti-TG6), and anti-gliadin. Anti-gliadin has become obsolete in the current diagnostic work up of CD, because of the poor test accuracy and the introduction anti-TG2 and anti-endomysium tests with a high sensitivity and specificity [10]. Anti-gliadin in the absence of anti-TG2 may occur in individuals with suspected non-coeliac gluten sensitivity. In addition, anti-gliadin and anti-TG6 have been associated with gluten ataxia and gluten neuropathy [11–13].

Gluten ataxia is the most extensively studied entity in GRND. Anti-TG2 IgA and/or anti-TG6 IgA deposits have been demonstrated in duodenal and brain tissue of patients with suspected gluten ataxia with and without enteropathy, which suggests a causative role for gluten [2, 3]. Large systematic studies into the effect of a gluten-free diet in patients with suspected gluten ataxia are lacking, but a small uncontrolled study showed significant improvement of symptoms and deficits compared to no intervention [4]. However, serological studies have not been conclusive. While most show a higher prevalence of elevated anti-gliadin and anti-TG6 in patients with sporadic ataxia compared to controls, others show no increased prevalence of gluten-related antibodies [2, 5, 6].

Two serological studies assessed the prevalence of gluten-related antibodies in patients with different forms of neuropathy and showed increased prevalence of anti-gliadin IgG or IgA compared to controls (32% vs 12%), and increased prevalence of elevated anti-TG2 IgA in patients with idiopathic neuropathy (7% vs 1%) and in chronic inflammatory demyelinating polyneuropathy (16% vs 1%) [7]. However, the neurological disease entities in these studies were not well defined and had small sample sizes. In a small unblinded non-randomised intervention study, the patients with “gluten neuropathy” reported improvement of symptoms after gluten-free diet [14]. Controlled studies for prevalence of gluten-related antibodies in patients with small fibre neuropathy (SNF) are lacking [9].

The aim of the current study was to further investigate a possible link between gluten sensitivity and cerebellar ataxia, SFN, and polyneuropathy in a large group of patients.

## Methods

### Design

We carried out a cross-sectional study to compare the prevalence of gluten-related antibodies in patients with idiopathic cerebellar ataxia, idiopathic SFN and chronic idiopathic axonal polyneuropathy (CIAP) to controls with a comparable age and women/men ratio to the whole disease group. All included individuals (*n* = 476 patients and *n* = 195 controls) were analysed for anti-gliadin IgA/IgG and anti-TG2 IgA. Additionally, analysis of anti-TG6 IgA/IgG was performed in all subjects of whom sufficient volume of serum was available (411patients and 194 controls). None of the included patients were treated with intravenous immunoglobulins or other immune modulating therapies. This was a multicentre study initiated by the Amsterdam UMC. This study was assessed by the local medical ethics committee, who waived the need for formal ethical review (case number 2018.396). Most data and samples were collected in the context of previously completed studies, and, when needed, additional written consent for this study was obtained.

### Patient groups

#### Idiopathic cerebellar ataxia

The adult patients referred with idiopathic cerebellar ataxia to the tertiary ataxia outpatient clinic of the Radboud University Medical Center in Nijmegen between 2019 and 2021 were included. Blood was drawn as a standard diagnostic procedure and was tested for anti-TG2 IgA, leftover material was used for further analysis in this study.

#### Idiopathic small fibre neuropathy

Patients with idiopathic SFN were diagnosed at the neurology outpatient clinic of the Maastricht University Medical Center. All consecutive patients seen between 2010 and 2015 that met the diagnostic criteria were included. To meet the criteria of SFN, the patients needed to have the typical complaints of SFN combined with a reduced intra-epidermal nerve fibre density in skin biopsy and/or abnormal temperature threshold testing without large nerve fibre involvement based on neurological examination (normal muscle strength, vibration sense, and tendon reflexes) and nerve conduction studies. Medical history was obtained in a standardised fashion, and blood and urine analysis and a chest X-ray were performed to exclude the following underlying conditions; alcohol abuse, diabetes mellitus including glucose intolerance, haemochromatosis, autoimmune diseases (including CD), monoclonal gammopathy of undetermined significance, sodium channel gene mutations, and vitamin B12 deficiency [15]. Blood was drawn as a standard diagnostic procedure, leftover material was used for further analysis in this study.

#### Chronic idiopathic axonal polyneuropathy

The patients with CIAP were diagnosed at the neurology outpatient clinic of the UMC Utrecht between 2008 and 2012. To be enrolled, patients had to meet inclusion criteria as previously described [16]. In short patients with a clinical diagnosis of polyneuropathy based on symptoms and neurological examination, nerve conduction studies ruling out demyelination and confirming large fibre involvement, exclusion of recognised causes of polyneuropathy by medical history, family history, and laboratory investigations [16]. Stored serum obtained in the context of the previously conducted case–control study was used for further analysis in this study.

#### Controls

We used clinical data and sera of healthy control subjects included in the prospective population-based amyotrophic lateral sclerosis study in the Netherlands (PAN) between 2008 and 2012 [17]. The selected controls were a subset of the total control population who gave informed consent for the use of blood samples for future research. The exclusion criteria were a known diagnosis of polyneuropathy, or a major risk factor for polyneuropathy such as excessive alcohol consumption (≥ 4 units per day), diabetes mellitus, or thyroid disease. Stored serum obtained in the context of the PAN study was used for further analysis in this study.

### Serological antibody detection

Serum was tested for anti-gliadin IgA/IgG (i.e. not anti-deamidated-gliadin), anti-TG2 IgA, and anti-TG6 IgA/IgG. The serum samples that were previously obtained were stored at −80 degrees Celsius until analyses were carried out. Antibodies against gliadin and TG2 were analysed using the PHADIA 250 fluorescent enzyme immunoassay (Thermo Fisher Scientific, Uppsala, Sweden). Serum samples were diluted 1:100 in the PHADIA instrument before measurement. For serum samples analysed on the PHADIA 250, our laboratory applies cut-off levels at 7.0 U/mL and 10.0 U/mL for anti-TG2 (IgA) and anti-gliadin, respectively. The lower cut-off for TG2 A (IgA) was chosen since it was previously demonstrated to improve the diagnostic test accuracy in adults [18]. For gliadin antibodies we also adopted a lower cut-off of 3.0 U/mL in addition to the manufacturer’s cut-off intended for the diagnosis of CD. This lower cut-off is based on observations in a cohort of gluten ataxia patients without enteropathy at a neurology outpatient clinic in Sheffield, as patients with a gliadin titre between 3 and 7 U/mL demonstrated both a clinical response and improvement on MR-spectroscopy after the initiation of a gluten-free diet [4, 19].

The samples were analysed for TG6 IgA and IgG antibodies using the ZediXclusive TG6-ab ELISA (IgA) and ZediXclusive TG6-ab ELISA (IgG) respectively (Zedira, Darmstadt, Germany). The samples and reagents were prepared according to manufacturer’s instruction. The serum samples were diluted 1:100. The advised cut-off values (4.0 U/mL for TG6 A IgA and 5.1 U/mL for TG6 A IgG) according to the Zedira TG6 kit were used. The positive and negative kit controls and biobank controls were included with every batch. All antigliadin and anti-TG6 antibody tests were conducted at the same location, with the same methodology using internal controls. For patients with SFN and cerebellar ataxia, anti-TG2 tests were performed as part of the standard diagnostic work up at the respective outpatient clinics. Since the laboratories that performed these tests also use the Phadia 250 test immune assay analyser to analyse anti-TG2, we did not perform a repeat anti-TG2 test, but included the original anti-TG2 results in this study.

### Statistical analysis

All statistical analyses were carried out using IBM SPSS Statistics for Windows version 26 (SPSS Inc., Chicago, Ill., USA. Age was reported as a mean and sex was reported in absolute numbers. Antibody positivity was described in absolute numbers and percentages. We analysed the number of seropositive patients and controls using the above mentioned cut-off values and compared differences in proportions using the chi square test or Fisher’s exact test in case one or more cell counts were less than 5. A binary logistic regression was used to model the association between antibody positivity (per antibody) and each condition separately (idiopathic cerebellar ataxia, SFN and CIAP), age and sex were included as variables in the model. A two-sided level of significance was set at *p* < 0.05.

### Data availability

Anonymized data not published within this article will be made available on request from any qualified investigator.

## Results

### Patient characteristics

A total of 476 patients and 195 controls were included. There was no significant difference in age and sex distributions overall between controls and patients (Table 1). In subgroup analysis, SFN patients were younger than controls, CIAP and idiopathic cerebellar ataxia patients were older than controls, SFN patients were more frequently female and CIAP patients were more frequently male than controls.

**Table 1:** Patient and control characteristics

	Women/Men (*n*)	Mean age in years (SD)	Minimal–maximal age
Controls	101/94	57 (9.9)	36–83
Disease groups	243/233	58 (13)	18–85
Idiopathic cerebellar ataxia	28/38	63 (12)	27–85
Idiopathic small fibre neuropathy	164/85	51 (12.1)	18–83
Chronic idiopathic axonal polyneuropathy	51/110	66 (9.5)	40–85

*SD* standard deviation

### The prevalence of gliadin antibodies

Using the manufacturer’s cut-off (10 U/mL) gliadin antibody seropositivity revealed no difference between patients and controls. Two patients had elevated anti-gliadin IgA (0.4%) versus one control (0.5%), five patients had elevated anti-gliadin IgG (1.1%) versus two controls (1.0%). One of these patients, with idiopathic cerebellar ataxia, also had elevated anti-TG2 IgA.

With the application of the lower cut-off for anti-gliadin of 3 U/mL, as described in the methods section, significant differences between disease and control group were observed. In the disease group 20.7% were positive for anti-gliadin IgA and 7.6% anti-gliadin IgG compared to 12.8% and 2.6% in the control group, respectively (*p* = 0.017 and *p* = 0.013).

In a subgroup analysis (Table 2), the patients with idiopathic cerebellar ataxia were more frequently positive for anti-gliadin IgG (> 3 U/mL) compared to controls with an odds ratio adjusted for age and sex of 8.9 (95% CI 2.84–27.72, *p* < 0.001 (Table 3). Patients with idiopathic SFN were more frequently positive for anti-gliadin IgA and IgG, the odds ratio adjusted for age and sex was only significant for anti-gliadin IgA, OR 3.51 (95%CI 2.03–6.04, *p* < 0.001). No differences were found between patients with CIAP and controls (Table 3).

**Table 2:** Prevalence of IgA and IgG antibodies against gliadin > 10 U/mL and > 3 U/mL

Group (*N*)	GA IgA > 10 U/mL *N* (%)	P value	GA IgA > 3 U/mL *N* (%)	*P* value	GA IgG > 10 U/mL *N* (%)	*P* value	GA IgG > 3 U/mL *N* (%)	*P* value
Controls (*n* = 195)	1 (0.5%)		25 (12.8%)		2 (1.0%)		5 (2.6%)	
Disease group (*n* = 476)	2 (0.4%)	1.000	98 (20.7%) *	**0.017**	5 (1.1%)	1.000	36 (7.6%) *	**0.013**
Idiopathic cerebellar ataxia (*n* = 66)	1 (1.5%)	0.443	10 (15.2%)	0.677	1 (1.5%)	1.000	12 (18.2%) *	** < 0.001**
Idiopathic small fibre neuropathy (*n* = 249)	0	0.441	66 (26.7%) *	**0.001**	4 (1.6%)	0.699	19 (7.6%) *	**0.020**
Chronic idiopathic axonal polyneuropathy (*n* = 61)	1 (0.6%)	1.000	22 (13.7%)	0.875	0	0.503	5 (3.1%)	0.760

*GA* gliadin antibodies

^*^Significant difference compared to controls with a *p*-value of < 0.050

**Table 3:** Logistic regression analysis

	Gliadin IgA (> 3U/ml)		Gliadin IgG (> 3U/ml)		TG6 IgA		TG6 IgG	
	OR	*P* value	OR	*P* value	OR	*P* value	OR	*P* value
Idiopathic cerebellar ataxia								
Unadjusted	1.214	0.631	**8.44**	** < 0.001**	2.173	0.055	0.978	0.967
Adjusted*	0.972	0.946	**8.88**	** < 0.001**	2.089	0.085	0.8244	0.727
Idiopathic small fibre neuropathy								
Unadjusted	**2.48**	** < 0.001**	**3.14**	**0.025**	1.66	0.098	0.874	0.715
Adjusted*	**3.51**	** < 0.001**	2.609	0.071	1.87	0.053	1.12	0.774
Chronic idiopathic axonal polyneuropathy								
Unadjusted	1.076	0.815	1.218	0.759	0.652	0.381	0.796	0.647
Adjusted	0.865	0.673	1.38	0.637	0.547	0.252	0.569	0.300

Significant difference compared to controls with a *p*-value of 0.050 are shown in bold

^*^For sex and age. *OR* odds ratio

### The prevalence of transglutaminase 6 antibodies

There was no difference in the seroprevalence of TG6 antibodies between patients and controls, which remained after adjusting for sex and age. (Tables 3 and 4).

**Table 4:** Prevalence of IgA and IgG antibodies against transglutaminase 6

Group (total)	Anti-TG6 IgA > 4.0 U/mL, *n* (%)	*p* value	Anti-TG6 IgG > 5.1 U/mL, *n* (%)	*p* value
Controls (*n* = 194)	18 (9.3%)		15 (7.7%)	
Disease group (*n* = 411)	54 (13.2%)	0.168	28 (6.8%)	0.681
Idiopathic cerebellar ataxia (*n* = 66)	12 (18.2%)	0.051	5 (7.6%)	0.967
Idiopathic small fibre neuropathy (*n* = 249)	36 (14.5%)	0.095	17 (6.8%)	0.715
Chronic idiopathic axonal polyneuropathy (*n* = 96)	6 (6.3%)	0.378	6 (6.3%)	0.647

*anti-TG6* transglutaminase 6 antibodies

### The prevalence of transglutaminase 2 antibodies

Three patients (0.6%) versus zero controls had elevated anti-TG2 levels, this difference was not significant. It is unknown whether these patients were diagnosed with CD. The first was a patient with idiopathic cerebellar ataxia, who tested positive for anti-TG2 IgA (238 U/mL), anti-gliadin IgA (28.0 U/mL), IgG (3.9 U/ml) and anti-TG6 IgA (8.0 U/mL). One SFN patient had an anti-TG2 IgA titre of 69.0 U/mL, and an anti-gliadin IgA titre of 6.6 U/mL, but had no seropositivity for anti-TG6. One CIAP patient had a low positive anti-TG2 IgA titre of 18 U/mL in absence of anti-gliadin or anti-TG6.

## Discussion

In the current study, we analysed the prevalence of gluten-related antibodies in a large group of patients with neurological disorders (idiopathic cerebellar ataxia, idiopathic SFN and CIAP), as compared to controls. The main conclusion is that there were no differences between disease groups and the control group for anti-gliadin, using the traditional cut-off (10 U/mL), nor for anti-TG6 or anti-TG2. Only when using a lower cut-off (3 U/mL), the disease group was more often positive for anti-gliadin than controls. Subgroup analysis with this low cut-off revealed that this was due to a higher prevalence of anti-gliadin IgG in the idiopathic cerebellar ataxia group and anti-gliadin IgA in the idiopathic SFN group.

The frequency of anti-gliadin levels > 10 U/mL in our study was much lower than previously described the general population (4–12%) in European and North American cohorts [5, 20–23]. The observed prevalence of 12% of anti-gliadin in the general population in other studies might be explained by selection bias, as patients tend to volunteer sooner for a seroprevalence study when they suffer from gastrointestinal symptoms [24]. This type of selection bias was avoided in the current study. The prevalence of anti-gliadin (manufacturer’s cut-off) observed in the idiopathic cerebellar ataxia group in this study was also lower than in several smaller studies (11.5–68%) [6, 22, 25, 26]. A difference in methodology of anti-gliadin testing and geographical region might also contribute to the large variability in seropositivity [27].

The significance of the increased frequency of low level anti-gliadin positivity in patients with SFN and idiopathic cerebellar ataxia compared to controls remains uncertain. The presence of low level gliadin antibodies might reflect a tendency to auto-immunity, not specifically addressed against gluten. Unfortunately, there was no data available on the presence of other antibodies, for example, antibodies directed against the peripheral or central nervous system, to further assess this theory. Anti-gliadin positivity has been demonstrated in up to 14% of CD-negative elderly and in patients with irritable bowel syndrome, suggesting a higher gut permeability as a cause of anti-gliadin positivity, without underlying pathology [28]. Increased gut permeability might induce paracellular gliadin peptide permeability and the formation of anti-gliadin antibodies. It is well recognised that an increased gut permeability and the microbiome play a significant role in multiple central nervous system disorders, such as Parkinson’s disease and MS [29]. This is further supported by the demonstration of higher prevalence of anti-gliadin seropositivity in other central nervous disorders, including hereditary spinocerebellar ataxia, amyotrophic lateral sclerosis and schizophrenia, compared to controls [27], 30 [31]. Interestingly, high prevalence of IgG and IgA antibodies against food antigens other than wheat gliadin such as hen’s egg albumen and cow’s milk lactoglobulin were observed in idiopathic ataxia and neuropathy [32], which might simply imply a higher gut permeability or a stronger immune response in general in these patients. The clinical importance of anti-gliadin is further questioned by the fact that 15% of patients previously diagnosed with gluten ataxia in a cohort later turned out to have repeat expansions in the RFC1 gene, leading to the alternative diagnosis of cerebellar ataxia, neuropathy and vestibular areflexia syndrome (CANVAS) [33]. Therefore, a possible correlation between anti-gliadin positivity and (extra-)intestinal symptoms should be interpreted with caution and only after excluding possible other causes.

In contrast to a prospective study showing seropositivity for TG6 in 49.6% of patients with unexplained cerebellar ataxia we did not find a difference in TG6 reactivity between patients and controls. Based on previous findings, Hadjivassiliou et al. have postulated that the pathological mechanism in GRND is partly explained by antibody cross-reactivity of transglutaminase 2 and 6 [2, 13]. Boscolo et al. demonstrated cross-reactivity of anti-TG2 IgA isolated from an untreated CD patient without known neurological complications with TG3 and TG6. [34]. Nevertheless, our data suggest that antibody cross-reactivity between TG2 and TG6 in humans does not play a significant role as anti-TG2 IgA were positive in only three individuals in our complete patient group as opposed to 73 TG6 IgA positive cases. Only one patient had both TG2 and TG6 antibodies. In a cohort of anti-TG6 seropositive CD patients with neurological complications, the anti-TG6 levels remained unchanged after the initiation of a gluten-free diet, contrary to anti-TG2 levels that decreased [35], while another study in gluten ataxia showed a drop in TG6 antibody levels after the initiation of a gluten-free diet [2]. It, therefore, remains to be established whether anti-TG6 reactivity is (directly or indirectly through TG2-cross-reactivity) gluten dependent. In literature, elevated anti-TG6 levels have mainly been demonstrated in disorders predominantly involving the central nervous system (including amyotrophic lateral sclerosis and multiple sclerosis), perhaps suggesting anti-TG6 as an a-specific marker of central nervous system pathology [2, 36, 37].

This study is unique because of its size and it is the first to compare the presence of gluten-related antibodies in idiopathic neurological disorders affecting the central as well as the peripheral nervous system to controls. A limitation is the largely retrospective nature, and lack of detailed information on medical history (especially for gastro-intestinal conditions) or dietary status. The patient groups and controls were included at different hospitals, which can be a potential cause for bias. However, the recruiting hospitals are tertiary centres with specialised outpatient clinics for these specific conditions, meaning that patients are referred from all over the Netherlands to these hospitals, limiting possible bias based on geographical location.

In conclusion, we did not find a higher prevalence of anti-gliadin in idiopathic cerebellar ataxia, CIAP and SFN versus healthy controls using the standard cut-off. Only when using a lower cut-off for anti-gliadin, a higher prevalence was observed for idiopathic ataxia and SNP versus controls. Whether to interpret a higher prevalence of low anti-gliadin titre as a causal effect or an epiphenomenon, remains unclear. No significant difference in the prevalence of anti-TG6 was observed for the included neurological disorders compared to controls. Based on our results, there does not appear to be a strong rationale for regular screening for gluten sensitivity in patients with idiopathic cerebellar ataxia, SFN and CIAP. The relevance of low titre anti-gliadin in idiopathic cerebellar ataxia and SFN, warrants further investigation.

## Abbreviations

anti-gliadin: Antibodies directed against gliadin
CIAP: Chronic idiopathic axonal polyneuropathy
CD: Coeliac disease
GRND: Gluten-related neurological diseases
NCS: Nerve conduction studies
OR: Odds ratio
PNP: Polyneuropathy
SFN: Small fibre neuropathy
Anti-TG2: Antibodies directed against transglutaminase 2
Anti-TG6: Antibodies directed against transglutaminase 6
